# Supplementary material for: ReLo is a simple and rapid colocalization assay to identify and characterize direct protein–protein interactions
Source: Nat Commun. 2024 Apr 3;15:2875. doi: 10.1038/s41467-024-47233-4 (PMC10991417; doi:10.1038/s41467-024-47233-4)

Supplementary Information to

ReLo is a simple and rapid colocalization assay to identify and characterize direct protein-protein interactions

Harpreet Kaur Salgania[1], Jutta Metz[1], Mandy Jeske[1]*

[1] Heidelberg University Biochemistry Center (BZH), Im Neuenheimer Feld 328, 69120 Heidelberg, Germany

Inventory:

Supplementary Figures 1-8

Supplementary Tables 1-3

Supplementary References

Source Data

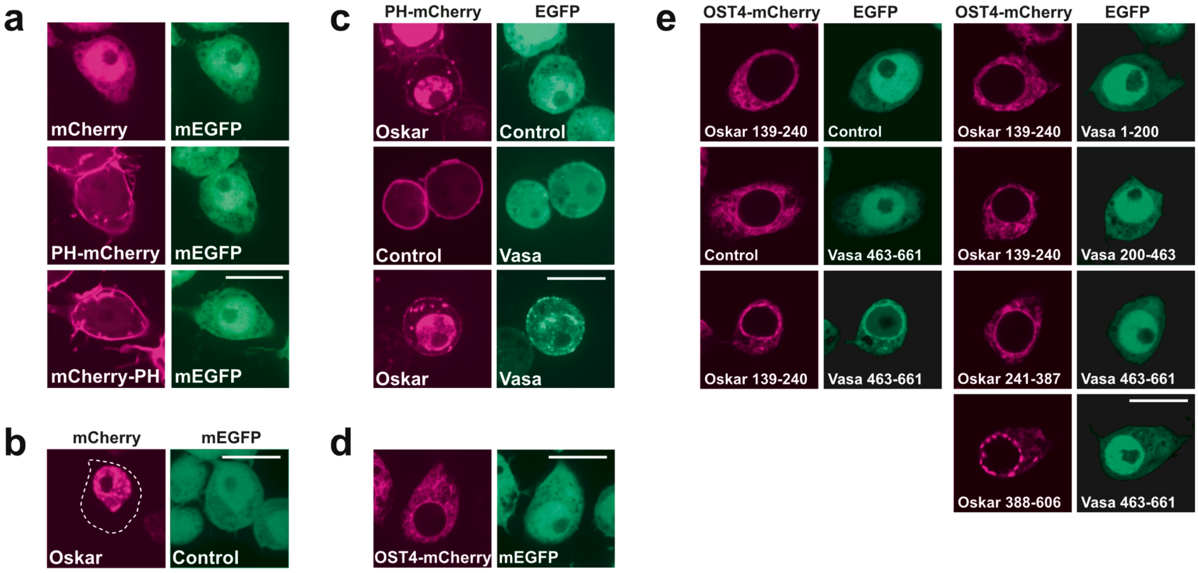

Supplementary Fig. 1. ReLo using the PH domain or the OST4 fusion.
mCherry, PH-mCherry, OST4-mCherry, or mEGFP alone or as fusions to the proteins indicated were coexpressed in S2R+ cells and their localization was analyzed by microscopy. **a** Both N- and C-terminal fusions to the PH domain directed the localization of mCherry to the plasma membrane. n=1 experiment. **b** mCherry-Oskar localized to the nucleus. n=4 experiments. **c** PH-mCherry Oskar retained in the nucleus and only partially localized to the plasma membrane (top panel). Vasa only partially relocalized with Oskar to the plasma membrane (bottom panel). n=2 experiments. **d** A fusion to OST4 directed the localization of mCherry to the ER. n=1 experiment; localization of OST4-fused construct is consistent with data shown in **Supplementary Fig. 1e.** and **Supplementary Fig. 4c.** **e** Vasa 463-661 but not Vasa 1-200 or 200-463 interacted with the Oskar eLOTUS domain (139-249). Vasa 463-661 did not interact with Oskar 241-396 or Oskar 398-606. n=1 experiment, which is consistent with the data shown in **Fig. 1c**. The scale bar is 10 µm.

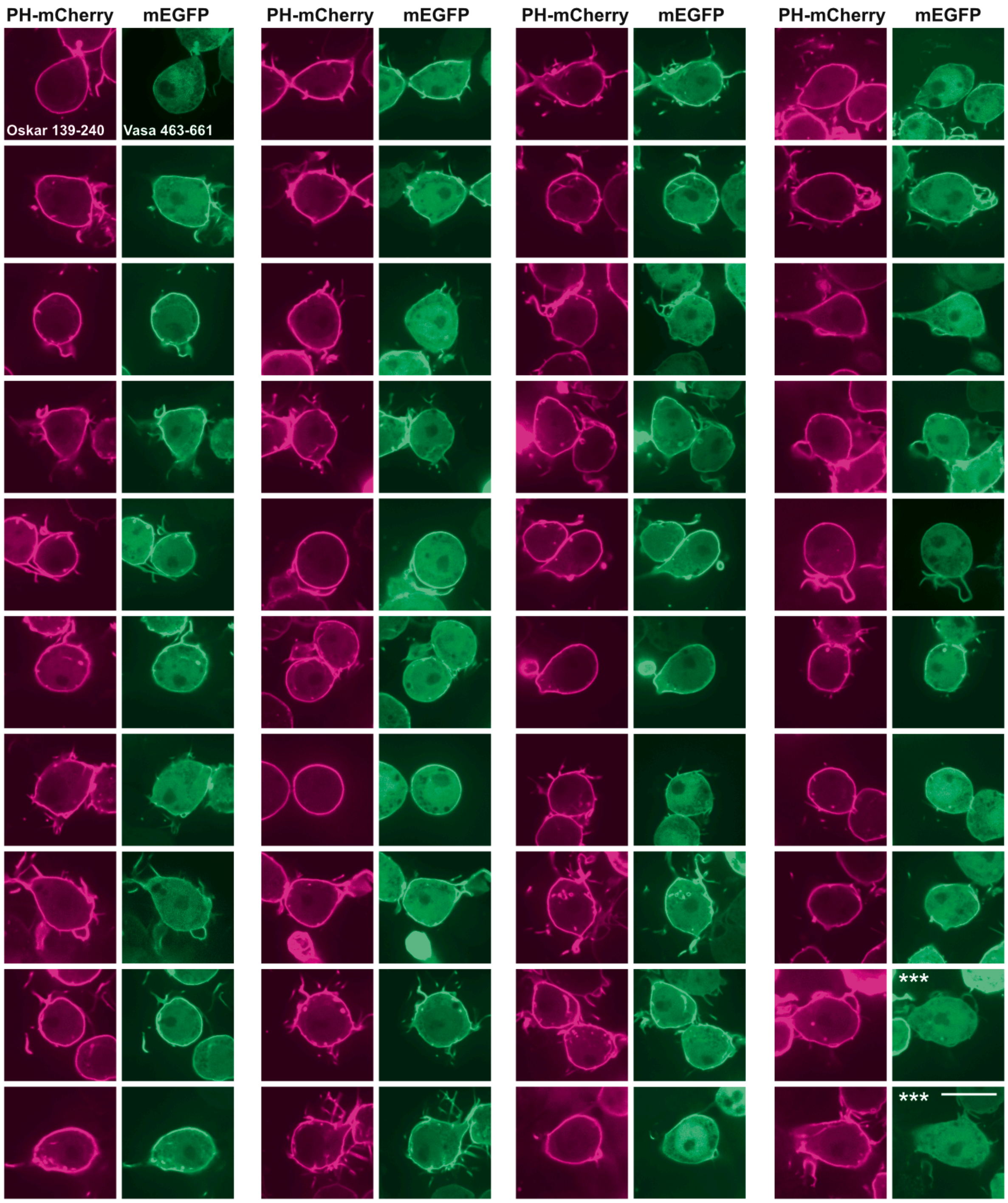

Supplementary Fig. 2. Assessing the Oskar-eLOTUS - Vasa-CTD interaction. PH-mCherry-Oskar 139-240 and mEGFP-Vasa 463-661 were coexpressed in S2R+ cells and their localization was analyzed by microscopy. In 38 of 40 (95%) cotransfected cells that were imaged, Vasa 463-661 relocalized to the plasma membrane in the presence of Oskar 139-240. ***, no obvious relocalization of mEGFP-Vasa 463-661 to the plasma membrane was detected, probably because the expression level of PH-mCherry-Oskar 139-240 was comparably low. In two additional replicates, we observed relocalization in 31 of 31 (100%) and in 22 of 23 (95%) cotransfected cells. n=3 experiments. The scale bar is 10 μm.

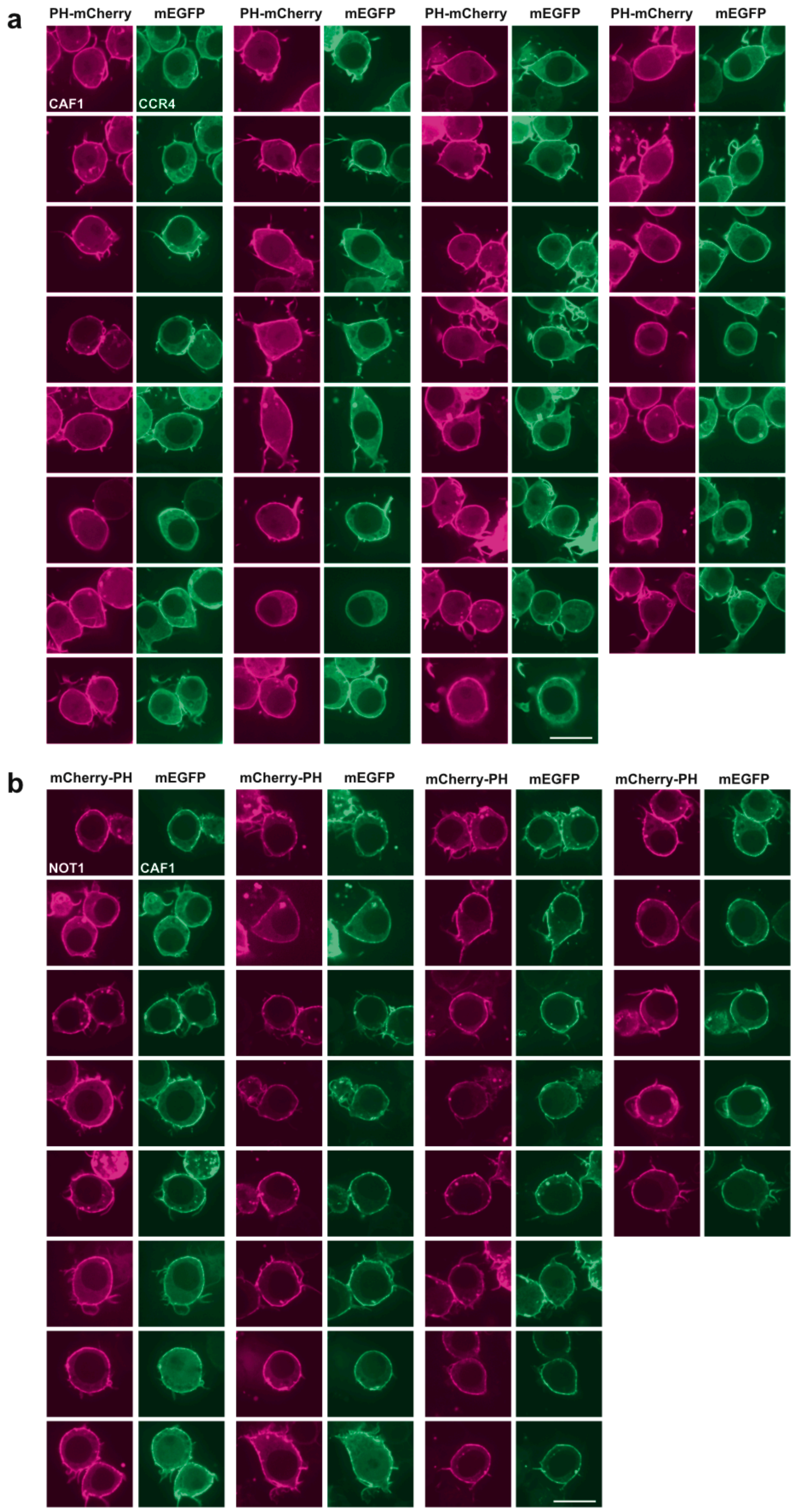

Supplementary Fig. 3. Assessing the CCR4-CAF1 and CAF1-NOT1 interactions. PH-mCherry, mCherry-PH, or mEGFP fusions to the proteins indicated were coexpressed in S2R+ cells and their localization was analyzed by microscopy. In 100% of the cells imaged an interaction between (**a**) CCR4 and CAF1 (31/31, 20/20, 45/45) or (**b**) CAF1 and NOT1 (29/29, 23/23, 30/30) was observed. n=3 experiments. The scale bar is 10 µm.

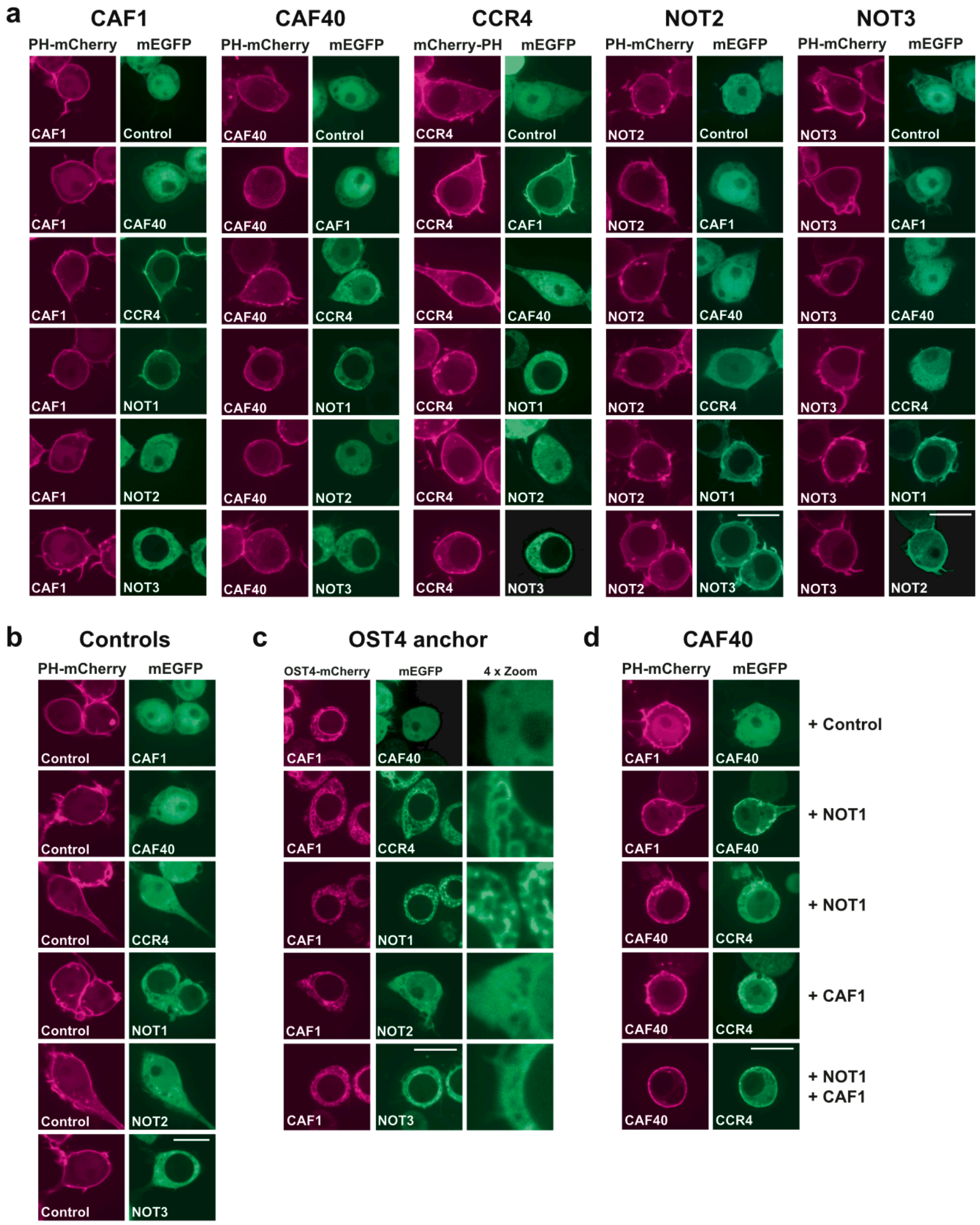

Supplementary Fig. 4. Interactions between subunits of the CCR4-NOT complex. PH-mCherry, OST4-mCherry, EGFP, or mEGFP fusions to the proteins indicated were coexpressed in S2R+ cells and their localization was analyzed by microscopy. **a** CAF1 recruited CCR4 and NOT1 to the plasma membrane but not CAF40, NOT2, or NOT3. CAF40 interacted with NOT1, and CCR4 interacted with CAF1. NOT2 bound to both NOT1 and NOT3. NOT3 interacted with both NOT1 and NOT2. **b** Localization of the core subunits of the CCR4-NOT complex fused to mEGFP in the presence of a PH-mCherry control plasmid. **c** PPIs between OST4 fusions of CAF1 and CCR4-NOT complex subunits. CAF1 recruited both CCR4 and NOT1 to the ER membrane, but not CAF40, NOT2, or NOT3. For data shown in **a-c,** n=1 experiment, which are consistent with each other, and consistent with data shown in **Fig. 3**. **d** CAF1 interacted with CAF40 upon NOT1 coexpression, and with CCR4 when both CAF1 and NOT1 were coexpressed. n=2 experiments. The scale bar is 10 µm.

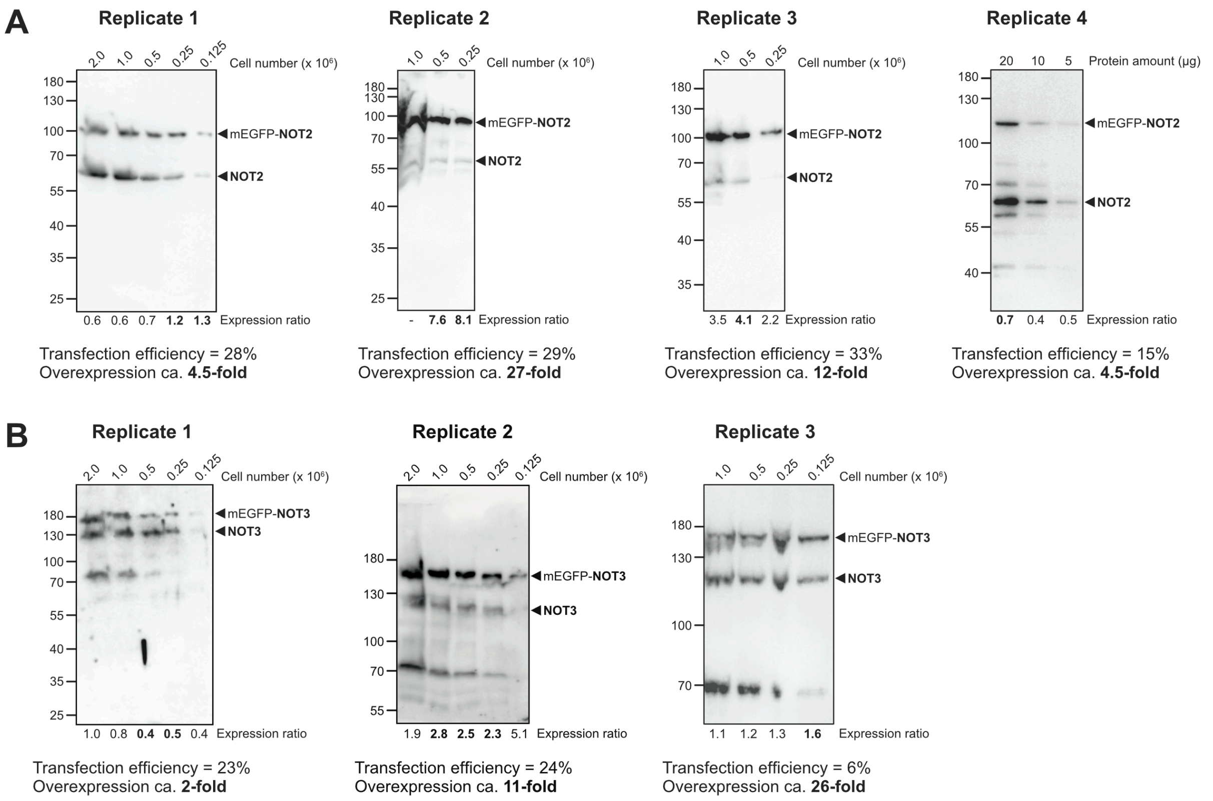

Supplementary Fig. 5. Protein expression levels of NOT2 and NOT3.
S2R+ cells expressing mEGFP-NOT2 (**a**) or mEGFP-NOT3 (**b**) were lysed and proteins analyzed by western blot using anti-NOT2 and anti-NOT3 antibodies, respectively. Signals were quantified using Fiji[1], and ratios of ectopically expressed to endogenous protein were calculated and are indicated below the blots. Ratios in bold were averaged and corrected for the transfection efficiency, which is indicated below the blots. Cell transfection efficiencies were determined by microscopy using Fiji prior to western blot analysis.

a

Subunit	Expression levels in S2R+ cells (RPKM)
Arp2	56
Arp3	329
Arpc1	263
Arpc2	97
Arpc3B	24
Arpc4	170
Arpc5	49
CAF1	28
CAF40	74
CCR4	16
NOT1	47
NOT2	48
NOT3	87

No - low (0-10)
Moderate (11-25)
Moderately high (26-50)
High (51-100)
Very high (101-1000)

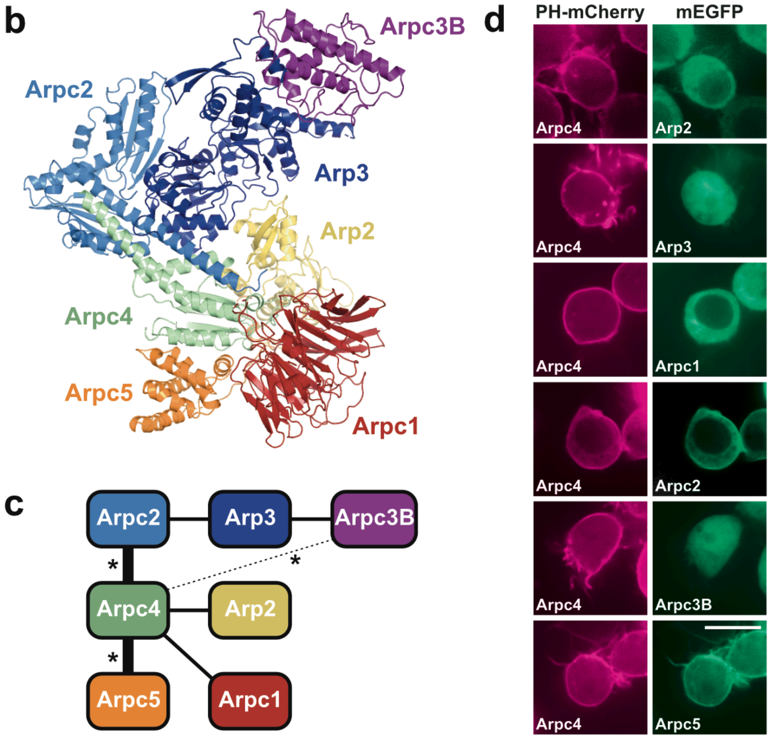

Supplementary Fig. 6. Interactions between subunits of the Arp2/3 complex.
a Expression levels of the subunits of the Arp2/3 and of the CCR4-NOT complex in S2R+ cells[2,3]. RPKM: reads per kilobase per million mapped bases. **b** Crystal structure of the bovine Arp2/3 complex (PDB ID:1K8K)[4]. **c** Scheme indicating the expected PPIs between the subunits of the Arp2/3 complex based on the crystal structure (straight connecting lines). Asterisks (*) indicate three interactions that were observed in pairwise Y2H tests with subunits of the human Arp2/3 complex[5]; of these three, the Arpc4 - Arpc3B interaction (dotted connecting line) is not expected based on the crystal structure. Connecting lines in bold represent PPIs that were observed in the ReLo assay. **d** PPI tests performed with the ReLo assay showing the results of the Arpc4 subunit, as an example. No interactions were observed when testing the subunits Arp2, Arp3, Arpc1, or Arpc3B (data not shown). PH-mCherry-Arpc4 and mEGFP fusions to the proteins indicated were coexpressed in S2R+ cells and their localization was analyzed by microscopy. Arpc4 interacted with Arpc2 and Arpc5. n=2 experiments. The scale bar is 10 µm.

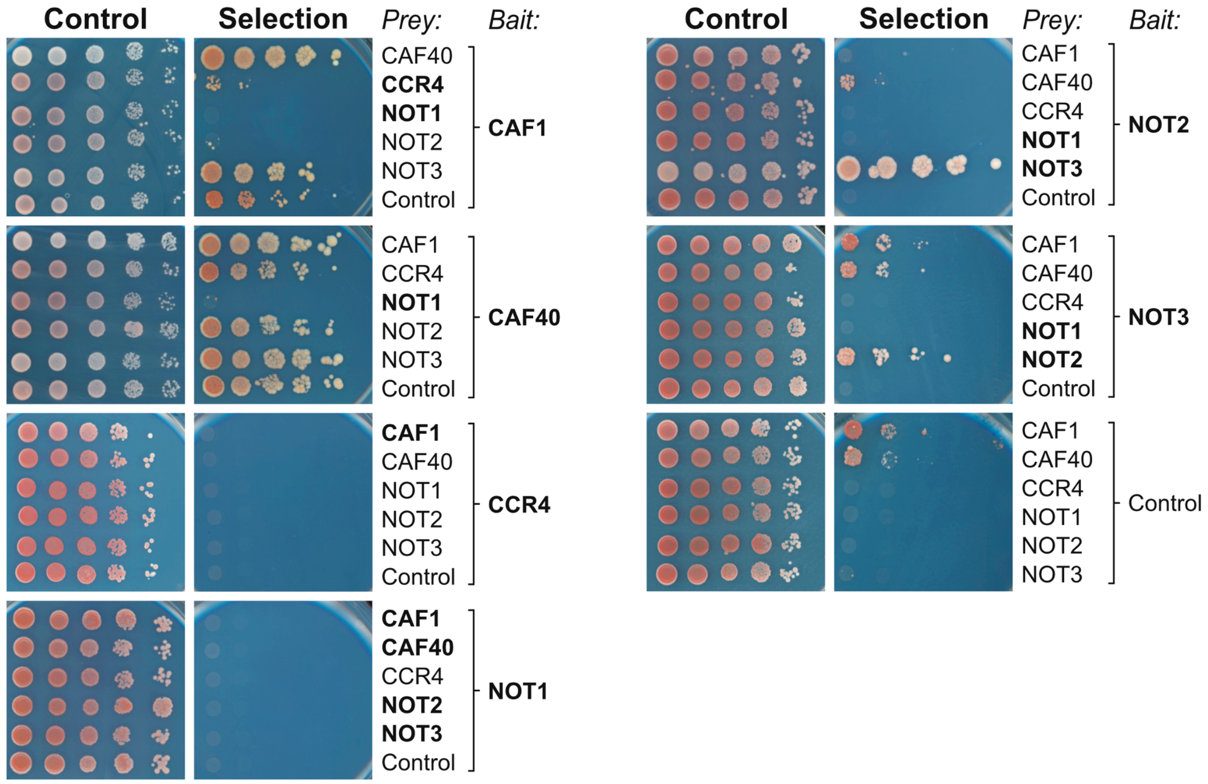

Supplementary Fig. 7. Interaction tests between the subunits of the CCR4-NOT complex using MYTH.
MYTH assays were performed with bait and prey constructs containing *Drosophila* proteins as indicated or no insertion (control plasmid). Five 10-fold dilutions of the cells were spotted and imaged after two (control plate) or six (selection plate) days of incubation. Selection medium lacked adenine and histidine. PPIs that were expected to be observed are indicated by protein names in bold letters. n=2 experiments for screening interactions with CAF1, CAF40, and CCR4 as baits, and n=3 experiments for screening interactions with NOT1, NOT2, and NOT3 as baits.

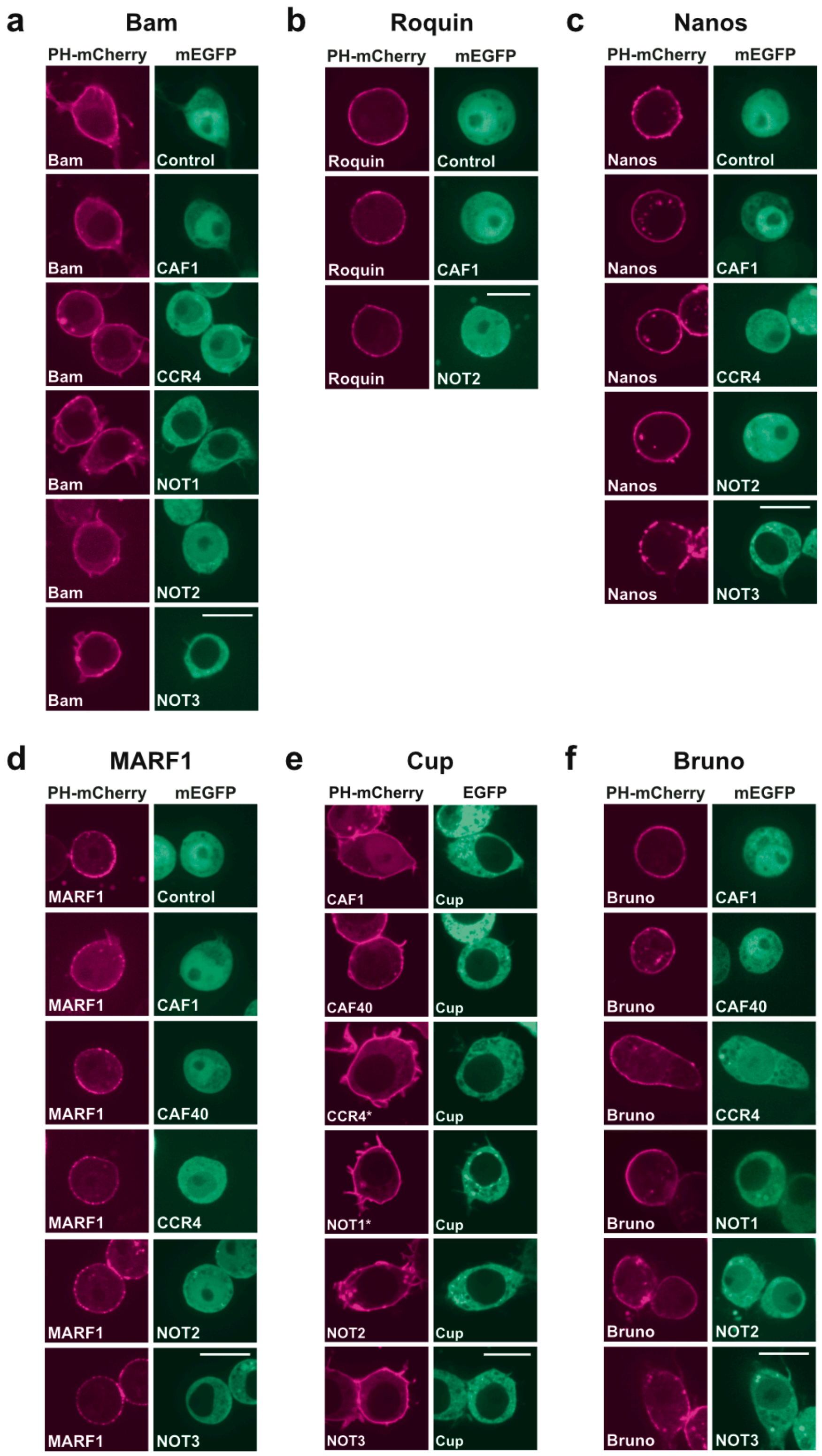

Supplementary Fig. 8. Negative results obtained from PPI tests between the subunits of the CCR4-NOT complex and repressor proteins.
PH-mCherry, mEGFP, or EGFP fusions to the proteins indicated were coexpressed in S2R+ cells and their localization was analyzed by microscopy. Shown are the negative results for the interaction tests between subunits of the CCR4-NOT complex and the proteins Bam (**a**), Roquin (**b**), Nanos, (**c**), MARF1 (**d**), Cup (**e**), and Bruno (**f**). *, CCR4 and NOT1 carried a C-terminal mCherry-PH fusion. For all, n=2 experiments. The scale bar is 10 µm.

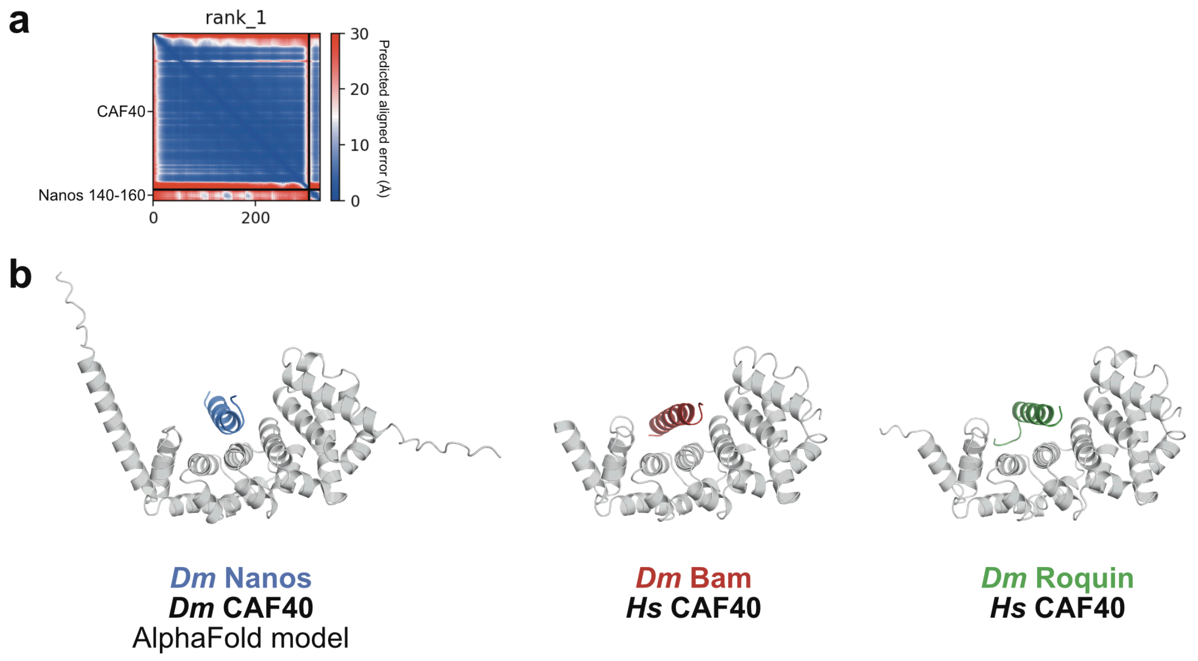

Supplementary Fig. 9. Comparison of the predicted structure of Nanos-CAF40 to experimental structures of CAF40-peptide complexes.

a Plot showing the predicted aligned error of the structural model obtained using AlphaFold-Multimer version 3 and depicted in **Fig. 4h**. **b** *Drosophila* (*Dm*) or human (*Hs*) CAF40 (grey color) as indicated bound to *Drosophila* Nanos (blue color; obtained using AlphaFold-Multimer version 3), *Drosophila* Bam (red color; PDB ID: 5ONB[6]) or *Drosophila* Roquin (green color; PDB ID: 5LSW[7])

Supplementary Table 1. Comparison of ReLo to other cell-based PPI methods.

	**ReLo**	**Y2H** [8] (classical yeast two-hybrid assay)	**MYTH** [9] (split-ubiquitin based membrane Y2H assay)	**MAPPIT, KISS** [10–12] (mammalian two-hybrid assays)	**PCA** (protein complementation assay)	**FRET** (Förster resonance energy transfer)
**Principle**	Bait and prey proteins are fused to different fluorescent proteins. In addition, the bait is anchored to a membrane. Upon interaction, the prey relocalizes to the bait.	Bait and prey proteins are fused to the GAL4 DNA-binding and activation domains, respectively. Upon the interaction, the GAL4 transcription factor (TF) is reconstituted, thereby activating the reporter gene(s).	Bait and prey proteins are fused to complementary ubiquitin fragments. In addition, the bait protein is anchored to the ER membrane and fused to a TF. Upon interaction the ubiquitin is reconstituted, which triggers proteolytic cleavage and release of the TF. The TF is imported into the nucleus and activates the transcription of a reporter gene.	**MAPPIT**<br>Bait and prey proteins are fused to signaling-deficient chimeric cytokine receptors. Upon interaction and subsequent ligand stimulation, the functional JAK-STAT signaling is restored, leading to the transcriptional activation of a reporter gene.<br><br>**KISS**<br>The bait protein is fused to the kinase domain of tyrosine kinase-2 (TYK2), and the prey is fused to the tyrosine motif-containing gp130 cytokine receptor fragment. Upon interaction, TYK2 phosphorylates the tyrosine residues in gp130, allowing recruitment of STAT3 and subsequent transcriptional activation of the reporter gene.	Bait and prey proteins are fused to complementary reporter fragments. Upon interaction, the functional reporter protein, such as an enzyme, a TF, or a fluorescent protein, is reconstituted. [13]	Bait and prey proteins are fused to donor and acceptor fluorophores, respectively. Upon interaction, the excited donor can transfer the energy to the acceptor fluorophore. [14]
**Readout**	Relocalization of the prey to the bait protein	Transcriptional activation of a reporter gene	Transcriptional activation of a reporter gene	Transcriptional activation of a reporter gene	Reconstitution of a fluorescent protein, enzyme, or TF	Light emission from the acceptor molecule
**Simultaneous monitoring of PPI and protein expression**	Yes	No: classical Y2H<br><br>Yes: tri-fluorescent Y2H [15]	No	No	No	Yes
**Testing bridging of PPIs**	Yes	Yes	Yes	Yes	No: in cases where the reconstitution of the split reporter fragments is sterically hindered<br><br>Yes: dual-color trimolecular fluorescence complementation [16]	No: classical FRET<br><br>Yes: three-chromophore FRET [17] and BiFC-based FRET [18]

	**ReLo**	**Y2H** [8] (classical yeast two-hybrid assay)	**MYTH** [9] (split-ubiquitin based membrane Y2H assay)	**MAPPIT, KISS** [10–12] (mammalian two-hybrid assays)	**PCA** (protein complementation assay)	**FRET** (Förster resonance energy transfer)
**Expression host**	*Drosophila* S2R+ cell line	*S. cerevisiae* strains	*S. cerevisiae* strains	Human embryonic kidney (HEK) 293 T cell line	Wide range of cell lines, tissues, and organisms	Wide range of cell lines, tissues, and organisms
**Possible challenges**<br><br>For all assays:<br><br>Tagging might sterically interfere with the PPI; testing both N- and C-terminal tagging might resolve the issue;<br><br>Possibility of unspecific interactions cannot be excluded in case of high overexpression	PH fusion may not completely localize nuclear proteins to the plasma membrane, and another anchor is required (e.g., OST4)<br><br>Transmembrane proteins with interacting regions within the lipid bilayer cannot be tested	Certain PPIs may require PTMs not present in yeast [19]<br><br>Membrane proteins, TFs, and proteins that cause autoactivation of the reporter gene cannot be tested [20,21]	Certain PPIs may require PTMs not present in yeast [19]<br><br>Instability of the protein fusions could release the transcription factor into the nucleus, leading to unwanted activation of the reporter genes [22,23]	Proteins that modulate the STAT3 signaling cannot be tested<br><br>Bait-independent interaction of prey with the cytokine receptor can occur with MAPPIT [24]<br><br>Transmembrane proteins cannot be tested with MAPPIT	Fusion to protein of interest may prevent the reconstitution of the split reporter fragments<br><br>Reconstitution of split reporter fragments due to high local concentrations (problematic for proteins with granular localization)<br><br>Reconstitution of split fluorescent protein fragments due to the inherent binding affinity towards each other [25–27]	Low signal-to-noise ratio [28,29]<br><br>FRET between adjacent non-interacting fluorophores due to high local concentrations [30,31]<br><br>Fusion to protein of interest may affect the dipole alignment of two fluorophores [32]
**Additional notes**	Human proteins have been tested with ReLo	Inexpensive experimental setup	Inexpensive experimental setup	Better assay sensitivity as compared to Y2H and PCA	Assay can be performed at physiological expression levels, which can be advantageous when testing toxic proteins [13]	Assay can be performed at physiological expression levels, which can be advantageous when testing toxic proteins [33,34]

Supplementary Table 2. DNA constructs used in this study.
All constructs are *Drosophila melanogaster* sequences, if not indicated otherwise. The specific protein isoforms (iso) used are indicated.

**Vector** (insertion site) (code)	**Final DNA construct**	**DNA template information**	**Code**
**pAc5.1-EGFP** (EcoRV) (T5-MJ)	pAc5.1-EGFP-**Aubergine** iso A	*Drosophila* ovarian cDNA	F20-MJ
	pAc5.1-EGFP-**Aubergine 4R→K (**R11K/R13K/R15K/R17K)	Site directed mutagenesis of pAc5.1-EGFP-Aubergine	HK121
	pAc5.1-EGFP-**Vasa** iso A	Reference: [35]	F15-MJ
	pAc5.1-EGFP-**Vasa 1-200**	pAc5.1-EGFP-Vasa	F17-MJ
	pAc5.1-EGFP-**Vasa 200-462**	pAc5.1-EGFP-Vasa	F18-MJ
	pAc5.1-EGFP-**Vasa 463-661**	pAc5.1-EGFP-Vasa	F19-MJ
	pAc5.1-EGFP-**Vasa 463-661 MUT** (F504E)	pAc5.1-EGFP-Vasa 463-661	HK196
	pAc5.1-EGFP-**Vasa closed** (E400Q)	Site directed mutagenesis of pAc5.1-EGFP-Vasa	F16-MJ
	pAc5.1-EGFP-**Vasa open** (K295N)	Site directed mutagenesis of pAc5.1-EGFP-Vasa	RR228
	pAc5.1-EGFP-**Vasa F504E**	Site directed mutagenesis of pAc5.1-EGFP-Vasa	HK197
	pAc5.1-EGFP-**Cup** iso B	pBSK-Cup-Flag (gift from Elmar Wahle)	F23-MJ
**pAc5.1-mEGFP** (EcoRV) (T6-MJ)	pAc5.1-mEGFP-**CAF1** iso A	pMTV5-Myc-CAF1 (gift from Elmar Wahle)[36]	HK50
	pAc5.1-mEGFP-**CAF40** iso A	pET19-CAF40 (gift from Elmar Wahle)	HK51
	pAc5.1-mEGFP-**CCR4** iso A	pMTV5-Myc-CCR4 (gift from Elmar Wahle)	HK52
	pAc5.1-mEGFP-**NOT1** iso D	pSPL_Strep_NOT1_NOT2 (gift from Elmar Wahle)	HK53
	pAc5.1-mEGFP-**NOT2** iso A	pSPL_Strep_NOT1_NOT2 (gift from Elmar Wahle)	HK54
	pAc5.1-mEGFP-**NOT3** iso A	pFL-Flag-NOT3 (gift from Elmar Wahle)	HK55
	pAc5.1-mEGFP-**human FRB**	pSF3-NBir-FKBP_CBir-FRB (gift from Julien Béthune)[37]	HK168
	pAc5.1-mEGFP-**human MDM2 1-118**	Addgene clone 70413 (gift from Dominic Esposito)	HK181
	pAc5.1-mEGFP-**Arp2** iso C	S2R+ cell cDNA	HK254
	pAc5.1-mEGFP-**Arp3** iso A	*Drosophila* testis cDNA	HK265
	pAc5.1-mEGFP-**Arpc1** iso A	*Drosophila* testis cDNA	HK268
	pAc5.1-mEGFP-**Arpc2** iso A	S2R+ cell cDNA	HK255

	pAc5.1-mEGFP-**Arpc3** iso C	S2R+ cell cDNA	HK256
	pAc5.1-mEGFP-**Arpc5** iso A	S2R+ cell cDNA	HK258
	pAc5.1-mEGFP-**CAF40 V186E**	pAc5.1-mEGFP-CAF40	HK275
**pAc5.1-mCherry** (EcoRV) (T7-MJ) (FspAI) (JM65)	pAc5.1-mCherry-**Oskar 139-606**	*oskar* cDNA	H2-MJ
	pAc5.1-mCherry-**Bruno** iso A	*bruno* cDNA	JM78
	pAc5.1-mCherry-**Nanos** iso B	*nanos* cDNA	JM154
**pAc5.1-λN-HA** (EcoRV) (T8-MJ)	pAc5.1-λN-HA-**CAF1** iso A	pMTV5-Myc-CAF1 (gift from Elmar Wahle)[36]	HK101
	pAc5.1-λN-HA-**NOT1** iso D	pSPL_Strep_NOT1_NOT2 (gift from Elmar Wahle)	JM155
	pAc5.1-λN-HA-**Cup** iso B	pBSK-Cup-Flag (gift from Elmar Wahle)	I14-MJ
**pAc5.1-PH-mEGFP** (FspAI) (JM50)	pAc5.1-PH-mEGFP-**Cup** iso B	pBSK-Cup-Flag (gift from Elmar Wahle)	JM51
	pAc5.1-PH-mEGFP-**Bruno** iso A	*bruno* cDNA	JM165
**pAc5.1-PH-mCherry** (FspAI) (HK49)	pAc5.1-PH-mCherry-**CAF1** iso A	pMTV5-Myc-CAF1 (gift from Elmar Wahle)[36]	HK96
	pAc5.1-PH-mCherry-**CAF40** iso A	pET19-CAF40 (gift from Elmar Wahle)	HK97
	pAc5.1-PH-mCherry-**NOT2** iso A	pSPL_Strep_NOT1_NOT2 (gift from Elmar Wahle)	HK99
	pAc5.1-PH-mCherry-**NOT3** iso A	pFL-Flag-NOT3 (gift from Elmar Wahle)	HK100
	pAc5.1-PH-mCherry-**human p53 1-50**	pcDNA3_1_3XHA_p53 WT (gift from Bernd Bukau)	HK180
	pAc5.1-PH-mCherry-**human FKBP12**	pSF3-NBir-FKBP_CBir-FRB (gift from Julien Béthune)[37]	HK174
	pAc5.1-PH-mCherry-**Oskar 139-606** iso A (Short Oskar)	pAc5.1-OST4-mCherry-Oskar 139-606	HK195
	pAc5.1-PH-mCherry-**Oskar 139-240** (eLOTUS domain)	pAc5.1-OST4-mCherry-Oskar 139-606	HK73
	pAc5.1-PH-mCherry-**Oskar 241-387** (DR)	pAc5.1-OST4-mCherry-Oskar 139-606	HK74
	pAc5.1-PH-mCherry-**Oskar 388-606** (OSK domain)	pAc5.1-OST4-mCherry-Oskar 139-606	HK75
	pAc5.1-PH-mCherry-**Oskar 139-240 MUT** (A162E/L228E)	Site directed mutagenesis of pAc5.1-PH-mCherry-Oskar 139-240	HK202
	pAc5.1-PH-mCherry-**Tudor** iso A	pAc5.1-EGFP-Tudor	HK130
	pAc5.1-PH-mCherry-**Bam** iso A	*Drosophila* ovarian cDNA	HK62

	pAc5.1-PH-mCherry-**Bam M24E**	Site directed mutagenesis of pAc5.1-PH-mCherry-Bam	HK88
	pAc5.1-PH-mCherry-**Nanos** iso B	*nanos* cDNA	HK65
	pAc5.1-PH-mCherry-**NanosΔ140-160**	pAc5.1 PH-mCherry-Nanos iso B	HK287
	pAc5.1-PH-mCherry-**Roquin** iso A	*Drosophila* ovarian cDNA	HK63
	pAc5.1-PH-mCherry-**MARF1** iso D	MARF1 cDNA[35]	HK56
	pAc5.1-PH-mCherry-**Bruno** iso A	*bruno* cDNA	JM72
	pAc5.1 PH-mCherry-**Arpc4** iso A	S2R+ cell cDNA	HK252
**pAc5.1-mCherry-PH** (FspAI) (EB3)	pAc5.1-**CCR4** (iso A)-mCherry-PH	pMTV5-Myc-CCR4 (gift from Elmar Wahle)	EB7
	pAc5.1-**NOT1** (iso D)-mCherry-PH	pSPL_Strep_NOT1_NOT2 (gift from Elmar Wahle)	EB5
**pAc5.1-OST4-mCherry** (EcoRV) (XH26)	pAc5.1-OST4-mCherry-**CAF1** iso A	pMTV5-Myc-CAF1(gift from Elmar Wahle)	HK184
	pAc5.1-OST4-mCherry-**Oskar 139-606** iso A	pAc5.1-mCherry-Oskar 139-606	H3-MJ
	pAc5.1-OST4-mCherry-**Oskar 241-387**	pAc5.1-mCherry-Oskar 139-606	HK193
	pAc5.1-OST4-mCherry-**Oskar 388-606**	pAc5.1-mCherry-Oskar 139-606	HK194
**pDHB1-MJ** (Eco47III) (JK16)	pDHB1-MJ-**CAF1** iso A	pMTV5-Myc-CAF1 (gift from Elmar Wahle)[6]	JK19
	pDHB1-MJ-**CAF40** iso A	pET19-CAF40 (gift from Elmar Wahle)	JK20
	pDHB1-MJ-**CCR4** iso A	pMTV5-Myc-CCR4 (gift from Elmar Wahle)	JK21
	pDHB1-MJ-**NOT1** iso D	pSPL_Strep_NOT1_NOT2 (gift from Elmar Wahle)	KM03
	pDHB1-MJ-**NOT2** iso A	pSPL_Strep_NOT1_NOT2 (gift from Elmar Wahle)	JK32
	pDHB1-MJ-**NOT3** iso A	pFL-Flag-NOT3 (gift from Elmar Wahle)	JK33
**pPR3-N-MJ** (SmaI) (JK18)	pPR3-N-MJ-**CAF1** iso A	Reference:[38]	JK76
	pPR3-N-MJ-**CAF40** iso A	Reference:[38]	JK77
	pPR3-N-MJ-**CCR4** iso A	Reference:[38]	JK78
	pPR3-N-MJ-**NOT1** iso D	pSPL_Strep_NOT1_NOT2 (gift from Elmar Wahle)	HK225
	pPR3-N-MJ-**NOT2** iso A	Reference:[38]	JK88
	pPR3-N-MJ-**NOT3** iso A	Reference:[38]	JK89

Sequence of the PH domain of *Rattus norvegicus* PLCδ:
HGLQDDPDLQALLKGSQLLKVKSSSWRRERFYKLQEDCKTIWQESRKVMRSPESQ LFSIEDIQEVRMGHRTEGLEKFARDIPEDRCFSIVFKDQRNTLDLIAPSPADAQHWV QGLRKIIHHSGSMDQRQK

Sequence of the *Saccharomyces cerevisiae* OST4 miniprotein:
MISDEQLNSLAITFGIVMMTLIVIYHAVDSTMSPKN

Linker sequences between fluorescence protein and protein of interest:
PSLNSATC (all ReLo vectors with both N-terminal tag and containing the PH sequence)
PSLNSAD (all other ReLo vectors with N-terminal tag)
ASSGGTN (all ReLo vectors with C-terminal tag)

Supplementary Table 3. Oligonucleotides used in this study.

Name	Sequence	Code	Plasmids generated with
Arp2 F	ATGGACAGCAAGGGTCGAAATG	HK-268	pAc5.1-mEGFP-Arp2 iso C
Arp2 R*	CTAGTGACTGATCTTTTGCAGCTTTTGC	HK-269	
Arp3 F	ATGGCAGGCAGGCTACCG	HK-270	pAc5.1-mEGFP-Arp3 iso A
Arp3 R*	TTATGTCATGGTGCCAAAGACGGG	HK-271	
Arpc1 F	ATGGCCGAGACATACACC	HK-272	pAc5.1-mEGFP-Arpc1 iso A
Arpc1 R*	CTAGATCTGCAGGTTGCGC	HK-273	
Arpc2 F	ATGATCCTGCTGGAAATCAATAATCGG	HK-274	pAc5.1-mEGFP-Arpc2 iso A
Arpc2 R*	TCAATCGATGCGCTTGAAAGTTC	HK-275	
Arpc3 F	ATGCCGGCCTACCACTCG	HK-276	pAc5.1-mEGFP-Arpc3 iso C
Arpc3 R*	TTATTGTCCAGGTCCAGCCAG	HK-277	
Arpc4 F	ATGGCAGCCACATTGAAGC	HK-278	pAc5.1 PH-mCherry-Arpc4 iso A
Arpc4 R*	CTAGAACCGTTTGAGGAACTCC	HK-279	
Arpc5 F	ATGGCCAAAAACACGTCCAG	HK-280	pAc5.1-mEGFP-Arpc5 iso A
Arpc5 R*	CTAGGCGCGATTTGTGTCGG	HK-281	
Aubergine F	ATGAATTTACCACCAAACCC	MJ-277	pAc5.1-EGFP-Aubergine iso A
Aubergine R*	TTACAAAAAGTACAATTGATTCTGCAG	MJ-278	
Aubergine R15K/R17K F	TAAGGGAAAGAAGCCCAATAATGTAGAGG	HK-92	pAc5.1-EGFP-Aubergine 4R-K (R11K/R13K/R15K/R17K)
Aubergine R11K/R13K R	CCCTTTCCCTTAGCAATTACAGGGTTTGG	HK-93	
Bam F	ATGCTTAATGCACGTGACGTG	HK-27	pAc5.1-PH-mCherry-Bam iso A
Bam R*	TTAGCTTCTGAAGCGAGGTACAC	HK-28	
Bam E25 F	GAGGAGCATTTGGCCTTAATGG	HK-68	pAc5.1-PH-mCherry-Bam M24E
Bam M24E R	TTCCTGCTTAAAATTGTGGTCCAAC	HK-69	
Bruno F	ATGTTCACCAGCCGCGCTTC	MJ-304	pAc5.1-mCherry-Bruno iso A, pAc5.1-PH-mEGFP-Bruno iso A, pAc5.1-PH-mCherry-Bruno iso A
Bruno R*	CTAGTAGGGCTTCGAGTCCTTG	JM-76	
CAF1 F	ATGAAATGGACAATGCCC	JK-54	pAc5.1-mEGFP-CAF1 iso A, pAc5.1-λN-HA-CAF1 iso A, pAc5.1-PH-mCherry-CAF1 iso A, pAc5.1-OST4-mCherry-CAF1 iso A, pDHB1-MJ-CAF1 iso A, pPR3-N-MJ-CAF1 iso A
CAF1 R	TGAAGCGCTGTTCGTC	JK-55	
CAF40 F	ATGAGTGCTCAACCGAG	JK-48	pAc5.1-mEGFP-CAF40 iso A, pAc5.1-PH-mCherry-CAF40 iso A, pDHB1-MJ-CAF40 iso A, pPR3-N-MJ-CAF40 iso A
CAF40 R	GGAGCCCAGTGGCGAC	JK-49	
CAF40 V186D F	GAGGCCACTTTCATCATCCAG	HK-288	pAc5.1-mEGFP-CAF40 V186E
CAF40 T185 R	AGTCTTGCTCAGCTCCGATC	HK-289	
CCR4 F	ATGAAAGGCAATCATTATAAAATG	JK-52	pAc5.1-mEGFP-CCR4 iso A, pAc5.1-CCR4 (iso A)-mCherry-PH, pDHB1-MJ-CCR4 iso A, pPR3-N-MJ-CCR4 iso A
CCR4 R	CCGGCGATTGATCAG	JK-53	
Cup F	ATGCAAATGGCCGAAGCTGAGC	MJ-271	pAc5.1-EGFP-Cup iso B, pAc5.1-λN-HA-Cup iso B, pAc5.1-PH-mEGFP-Cup iso B
Cup R*	TTAATGAAACTCATCCCCGCTGTTG	MJ-272	
Human FKBP F	ATGGGAGTGCAGGTGGAAAC	HK-181	pAc5.1-PH-mCherry-human FKBP12
Human FKBP R*	CTATTCCAGTTTTAGAAGCTCCAC	HK-182	
Human FRB F	ATGATCCTCTGGCATGAGATG	HK-179	pAc5.1-mEGFP-human FRB
Human FRB R*	CTACTTTGAGATTCGTCGGAACAC	HK-180	
Human MDM2 F	ATGGTGAGGAGCAGGCAAATG	HK-173	pAc5.1-mEGFP-human MDM2 1-118
Human MDM2 Q118 R*	CTACTGATTGACTACTACCAAGTTCCTG	HK-175	
Human p53 F	ATGGAGGAGCCGCAGTCAG	HK-176	pAc5.1-PH-mCherry-human p53 1-50
Human p53 I50 R*	CTAAATATCGTCCGGGGACAG	HK-178	
MARF1 F	ATGTTTCAGCGACACACGTC	MJ-296	pAc5.1-PH-mCherry-MARF1 iso D
MARF1 R	AGCTATTTTACGATCGTGATTATC	BZH-121	
Nanos F	ATGTTCCGCAGCAACTTGG	HK-31	pAc5.1-mCherry-Nanos iso B, pAc5.1-PH-mCherry-Nanos iso B
Nanos R*	CTAAACCTTCATCTGTTGCTTG	HK-32	
Nanos D161 F	GATCTCGGTCGCATGTCCTACG	HK-302	pAc5.1-PH-mCherry-NanosΔ140-160
Nanos E139 R	TTCTGCTGCGCCCGTCGTC	HK-303	
NOT1 F	ATGAACGTAGAGAGCCAACTG	JK-46	pAc5.1-mEGFP-NOT1 iso D, pAc5.1-λN-HA-NOT1 iso D, pAc5.1-NOT1 (iso D)-mCherry-PH, pDHB1-MJ-NOT1 iso D, pPR3-N-MJ-NOT1 iso D
NOT1 R	GTTGATGGTGGCTACC	JK-47	

Name	Sequence	Code	Plasmids generated with
NOT2 F	ATGGCGAATTTAAATTTTCAAC	JK-58	pAc5.1-mEGFP-NOT2 iso A, pAc5.1-PH-mCherry-NOT2 iso A, pDHB1-MJ-NOT2 iso A, pPR3-N-MJ-NOT2 iso A
NOT2 R	TACAGACTGTCCATTCATAAAC	JK-59	
NOT3 F	ATGGCTGCGACGAGAAAATTG	JK-50	pAc5.1-mEGFP-NOT3 iso A, pAc5.1-PH-mCherry-NOT3 iso A, pDHB1-MJ-NOT3 iso A, pPR3-N-MJ-NOT3 iso A
NOT3 R	ATTCAGCTCCTTGTCCTC	JK-51	
sOskar F	ATGACCATCATCGAGAGC	JM-64	pAc5.1-mCherry-Oskar 139-606, pAc5.1-PH-mCherry-Oskar 139-606 iso A (Short Oskar), pAc5.1-OST4-mCherry-Oskar 139-606 iso A
sOskar R*	TTAATACTCCAGGCTCGTTTC	JM-42	
sOskar F	ATGACCATCATCGAGAGC	JM-64	pAc5.1-PH-mCherry-Oskar 139-240 (eLOTUS domain)
Oskar S240 R	GCTGGTGCGCTCTTTCTGG	MJ-314	
Oskar A162E F	GAGATATTGCTGAGCCACGCC	HK-192	pAc5.1-PH-mCherry-Oskar 139-240 MUT (A162E/L228E)
Oskar R161 R	GCGCACCTCACTATCTATATCG	HK-193	
Oskar 241F	GACTACAGCAGCGGAGCTC	MJ-312	pAc5.1-PH-mCherry-Oskar 241-387 (DR), pAc5.1-OST4-mCherry-Oskar 241-387
Oskar M387 R*	TTACATTATGTTCATGCCGTTAAATGG	JM-352	
Oskar L388 F	ATGAAGAGACGCCACGAAATGAC	HK-40	pAc5.1-PH-mCherry-Oskar 388-606 (OSK domain), pAc5.1-OST4-mCherry-Oskar 388-606
sOskar R*	TTAATACTCCAGGCTCGTTTC	JM-42	
OST4 F	ATGATCTCTGATGAACAGCTG	HK-188	pAc5.1-OST4-mCherry (EcoRV)
OST4 R	CTAGTTCTTAGGAGACATGGTGG	HK-189	
PLC_PH_ F (SG)	GGCTCCATGCACGGTCTCCAGGATG	HK-128	pAc5.1-mCherry (FspAI) pAc5.1-mCherry-PH (FspAI)
PLC_PH R*	TTATTTCTGACGCTGGTCCATAG	HK-129	
FspAI SSG F	CCAGCGGCGGTACCAACATGGTGAGCAAGG	HK-126	
FspAI R	ATGCGCACCCGATCCGGGGTCTCTG	HK-127	
Q5 PLC delta PH F	GATCGGTACCAACATGCACGGTCTCCAGGA TGATCC	HK-5	pAc5.1-PH-mCherry (FspAI), pAc5.1-PH-mEGFP (FspAI)
Q5 PLC delta PH R	GATCGGTACCTTTCTGACGCTGGTCCATAG	HK-6	
fspAI F	GCATGAGATATCCAGCACAGTGGC	HK-19	
fspAI R	GCACGTTGCAGAATTCAAGCTTGG	HK-20	
Roquin F	ATGCCGATTCAGGCTCCC	HK-29	pAc5.1-PH-mCherry-Roquin iso A
Roquin R*	CTAGTCCACCTTGATCCCAC	HK-30	
Tudor F	ATGAATGGACAGGCGCGC	MJ-291	pAc5.1-PH-mCherry-Tudor iso A
Tudor R*	TCACTGACATTCCTGAAGC	MJ-292	
Vasa F	ATGTCTGACGACTGGGATG	MJ-267	pAc5.1-EGFP-Vasa iso A
Vasa R*	TCAATCCCATTGCTCTTCTTC	MJ-268	

Supplementary References

1. Schindelin, J. *et al.* Fiji: An open-source platform for biological-image analysis. *Nat Methods* **9**, 676–682 (2012).
2. Brown, J. B. *et al.* Diversity and dynamics of the Drosophila transcriptome. *Nature 2014 512:7515* **512**, 393–399 (2014).
3. Hu, Y., Comjean, A., Perrimon, N. & Mohr, S. E. The Drosophila Gene Expression Tool (DGET) for expression analyses. *BMC Bioinformatics* **18**, 98 (2017).
4. Robinson, R. C. *et al.* Crystal Structure of Arp2/3 Complex. *Science (1979)* **294**, 1679–1684 (2001).
5. Zhao, X., Yang, Z., Qian, M. & Zhu, X. Interactions among subunits of human Arp2/3 complex: p20-Arc as the hub. *Biochem Biophys Res Commun* **280**, 513–517 (2001).
6. Sgromo, A. *et al.* Drosophila Bag-of-marbles directly interacts with the CAF40 subunit of the CCR4-NOT complex to elicit repression of mRNA targets. *RNA* **24**, 381–395 (2018).
7. Sgromo, A. *et al.* A CAF40-binding motif facilitates recruitment of the CCR4-NOT complex to mRNAs targeted by Drosophila Roquin. *Nat Commun* **8**, 14307 (2017).
8. Fields, S. & Song, O. K. A novel genetic system to detect protein-protein interactions. *Nature* **340**, 245–246 (1989).
9. Stagljar, I., Korostensky, C., Johnsson, N. & Te Heesen, S. A genetic system based on split-ubiquitin for the analysis of interactions between membrane proteins in vivo. *Proc Natl Acad Sci U S A* **95**, 5187–5192 (1998).
10. Eyckerman, S. *et al.* Design and application of a cytokine-receptor-based interaction trap. *Nat Cell Biol* **3**, 1114–1119 (2001).
11. Lemmens, I., Lievens, S. & Tavernier, J. MAPPIT, a mammalian two-hybrid method for in-cell detection of protein-protein interactions. *Methods Mol Biol* **1278**, 447–455 (2015).
12. Masschaele, D., Gerlo, S., Lemmens, I., Lievens, S. & Tavernier, J. KISS: A Mammalian Two-Hybrid Method for In Situ Analysis of Protein-Protein Interactions. *Methods Mol Biol* **1794**, 269–278 (2018).
13. Blaszczak, E., Lazarewicz, N., Sudevan, A., Wysocki, R. & Rabut, G. Protein-fragment complementation assays for large-scale analysis of protein-protein interactions. *Biochem Soc Trans* **49**, 1337–1348 (2021).
14. Fang, C., Huang, Y. & Zhao, Y. Review of FRET biosensing and its application in biomolecular detection. *Am J Transl Res* **15**, 694–709 (2023).
15. Cluet, D. *et al.* A Quantitative Tri-fluorescent Yeast Two-hybrid System: From Flow Cytometry to In cellula Affinities. *Molecular & Cellular Proteomics* **19**, 701–715 (2020).
16. Offenborn, J. N., Waadt, R. & Kudla, J. Visualization and translocation of ternary Calcineurin-A/Calcineurin-B/Calmodulin-2 protein complexes by dual-color trimolecular fluorescence complementation. *New Phytologist* **208**, 269–279 (2015).
17. Galperin, E., Verkhusha, V. V & Sorkin, A. Three-chromophore FRET microscopy to analyze multiprotein interactions in living cells. *Nat Methods* **1**, 209–217 (2004).
18. Shyu, Y. J., Suarez, C. D. & Hu, C.-D. Visualization of AP-1–NF-κB ternary complexes in living cells by using a BiFC-based FRET. *Proceedings of the National Academy of Sciences* **105**, 151–156 (2008).

19. Coates, P. & Hall, P. The yeast two-hybrid system for identifying protein–protein interactions. *J Pathol* **199**, 4–7 (2003).
20. Dreze, M. *et al.* High-Quality Binary Interactome Mapping. in 281–315 (2010). doi:10.1016/S0076-6879(10)70012-4.
21. Flajolet, M. *et al.* A genomic approach of the hepatitis C virus generates a protein interaction map. *Gene* **242**, 369–379 (2000).
22. Lalonde, S. *et al.* Molecular and cellular approaches for the detection of protein-protein interactions: Latest techniques and current limitations. *Plant Journal* **53**, 610–635 (2008).
23. Xing, S., Wallmeroth, N., Berendzen, K. W. & Grefen, C. Techniques for the Analysis of Protein-Protein Interactions in Vivo. *Plant Physiol* **171**, 727–758 (2016).
24. Tavernier, J. *et al.* 256 : MAPPIT and Co: The importance of system artifacts. *Cytokine* **63**, 303–304 (2013).
25. Horstman, A., Tonaco, I. A. N., Boutilier, K. & Immink, R. G. H. A cautionary note on the use of split-YFP/BiFC in plant protein-protein interaction studies. *Int J Mol Sci* **15**, 9628–9643 (2014).
26. Kodama, Y. & Hu, C. D. Bimolecular fluorescence complementation (BiFC): a 5-year update and future perspectives. *Biotechniques* **53**, 285–298 (2012).
27. Kudla, J. & Bock, R. Lighting the way to protein-protein interactions: Recommendations on best practices for bimolecular fluorescence complementation analyses. *Plant Cell* **28**, 1002–1008 (2016).
28. Leavesley, S. J. & Rich, T. C. Overcoming limitations of FRET measurements. *Cytometry A* **89**, 325–327 (2016).
29. Piston, D. W. & Kremers, G. J. Fluorescent protein FRET: the good, the bad and the ugly. *Trends Biochem Sci* **32**, 407–414 (2007).
30. Broussard, J. A., Rappaz, B., Webb, D. J. & Brown, C. M. Fluorescence resonance energy transfer microscopy as demonstrated by measuring the activation of the serine/threonine kinase Akt. *Nat Protoc* **8**, 265–281 (2013).
31. Vogel, S. S., Thaler, C. & Koushik, S. V. Fanciful FRET. *Science's STKE* **2006**, (2006).
32. Broussard, J., B, R., DJ, W. & CM, B. Fluorescence resonance energy transfer microscopy of the serine/threonine kinase Akt. *Nat Protoc* **8**, 265–281 (2013).
33. Liang, Y. *et al.* TR-FRET Assays for Endogenous Huntingtin Protein Level in Mouse Cells. *J Huntingtons Dis* **3**, 253–259 (2014).
34. Sheng, L. *et al.* Imaging specific newly synthesized proteins within cells by fluorescence resonance energy transfer. *Chem Sci* **8**, 748–754 (2017).
35. Jeske, M., Müller, C. W. & Ephrussi, A. The LOTUS domain is a conserved DEAD-box RNA helicase regulator essential for the recruitment of Vasa to the germ plasm and nuage. *Genes Dev* **31**, 939–952 (2017).
36. Temme, C. *et al.* Subunits of the Drosophila CCR4-NOT complex and their roles in mRNA deadenylation. *RNA* **16**, 1356–70 (2010).
37. Schopp, I. M. *et al.* Split-BioID a conditional proteomics approach to monitor the composition of spatiotemporally defined protein complexes. *Nat Commun* **8**, 15690 (2017).
38. Pekovic, F. *et al.* RNA binding proteins Smaug and Cup induce CCR4–NOT-dependent deadenylation of the *nanos* mRNA in a reconstituted system. *Nucleic Acids Res* **51**, 3950–3970 (2023).

SOURCE DATA

Supp. Fig. 5A - replicate 1

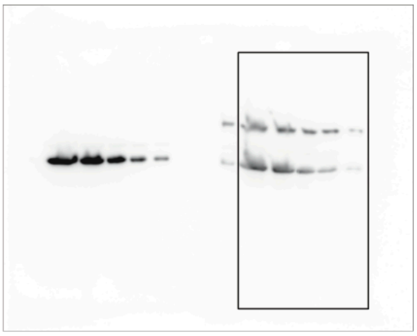

Supp. Fig. 5A - replicate 2

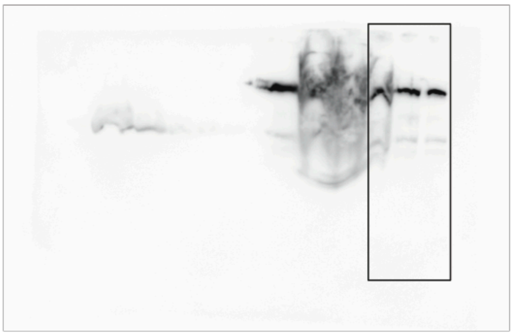

Supp. Fig. 5A - replicate 3

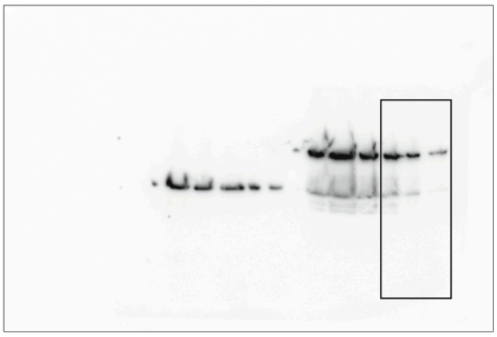

Supp. Fig. 5A - replicate 4

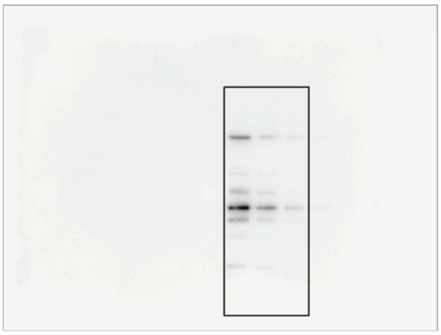

Supp. Fig. 5B - replicate 1

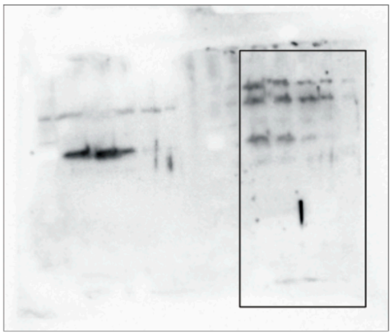

Supp. Fig. 5B - replicate 2

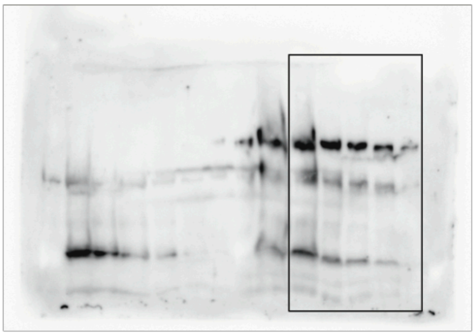

Supp. Fig. 5B - replicate 3

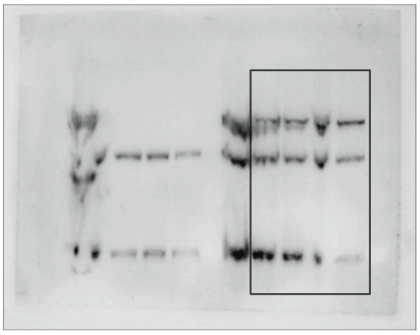

Supplement: Supplementary file 1 — Supplementary Information [file 41467_2024_47233_MOESM1_ESM.pdf]
