# Supplementary material for: ReLo is a simple and rapid colocalization assay to identify and characterize direct protein–protein interactions
Source: Nat Commun. 2024 Apr 3;15:2875. doi: 10.1038/s41467-024-47233-4 (PMC10991417; doi:10.1038/s41467-024-47233-4)
Supplement: Supplementary file 3 — Reporting Summary [file 41467_2024_47233_MOESM3_ESM.pdf]

nature portfolio

Corresponding author(s): Mandy Jeske

Last updated by author(s): Mar 8, 2024

Reporting Summary

Nature Portfolio wishes to improve the reproducibility of the work that we publish. This form provides structure for consistency and transparency in reporting. For further information on Nature Portfolio policies, see our Editorial Policies and the Editorial Policy Checklist.

Statistics

For all statistical analyses, confirm that the following items are present in the figure legend, table legend, main text, or Methods section.

n/a	Confirmed	
☐	☒	The exact sample size (*n*) for each experimental group/condition, given as a discrete number and unit of measurement
☒	☐	A statement on whether measurements were taken from distinct samples or whether the same sample was measured repeatedly
☒	☐	The statistical test(s) used AND whether they are one- or two-sided<br>*Only common tests should be described solely by name; describe more complex techniques in the Methods section.*
☒	☐	A description of all covariates tested
☒	☐	A description of any assumptions or corrections, such as tests of normality and adjustment for multiple comparisons
☒	☐	A full description of the statistical parameters including central tendency (e.g. means) or other basic estimates (e.g. regression coefficient) AND variation (e.g. standard deviation) or associated estimates of uncertainty (e.g. confidence intervals)
☒	☐	For null hypothesis testing, the test statistic (e.g. *F*, *t*, *r*) with confidence intervals, effect sizes, degrees of freedom and *P* value noted<br>*Give P values as exact values whenever suitable.*
☒	☐	For Bayesian analysis, information on the choice of priors and Markov chain Monte Carlo settings
☒	☐	For hierarchical and complex designs, identification of the appropriate level for tests and full reporting of outcomes
☒	☐	Estimates of effect sizes (e.g. Cohen's *d*, Pearson's *r*), indicating how they were calculated

Our web collection on statistics for biologists contains articles on many of the points above.

Software and code

Policy information about availability of computer code

Data collection	Microscopy images were taken with the microscopes and cameras as indicated in the methods section as follows: images of live cells were captured with either a Nikon Plan Apoλ 100x NA 1.45 oil objective and a Perkin Elmer Ulraview VoX System using a Yokogawa X-1 spinning disk scanhead (Hamamatsu C9100-50 camera, 840 x 1000 resolution), a Nikon Plan Apoλ 100x NA 1.45 oil objective and Perkin Elmer Ulraview VoX System using a Yokogawa X-1 spinning disk scanhead (Hamamatsu C9100-02 EMCCD camera, 1000 x 1000 resolution), or a Nikon Apo 60x NA 1.40 oil-λS objective and a Nikon Eclipse Ti2 inverted microscope (Nikon confocal Ax camera, 1024 x 1024 resolution). The acquisitions software was Volocity for the first two microscopes and NIS elements AR (version 5.41.02) for the latter.
Data analysis	Images were analyzed and processed with Fiji software. (ImageJ version 1.53t); Pymol (version 2.4.0)

For manuscripts utilizing custom algorithms or software that are central to the research but not yet described in published literature, software must be made available to editors and reviewers. We strongly encourage code deposition in a community repository (e.g. GitHub). See the Nature Portfolio guidelines for submitting code & software for further information.

Data

Policy information about availability of data

All manuscripts must include a data availability statement. This statement should provide the following information, where applicable:

- Accession codes, unique identifiers, or web links for publicly available datasets
- A description of any restrictions on data availability
- For clinical datasets or third party data, please ensure that the statement adheres to our policy

The authors declare that all relevant data supporting the findings of this study are available within the article and its supplementary information files.

Research involving human participants, their data, or biological material

Policy information about studies with human participants or human data. See also policy information about sex, gender (identity/presentation), and sexual orientation and race, ethnicity and racism.

Reporting on sex and gender	N/A
Reporting on race, ethnicity, or other socially relevant groupings	N/A
Population characteristics	N/A
Recruitment	N/A
Ethics oversight	N/A

Note that full information on the approval of the study protocol must also be provided in the manuscript.

Field-specific reporting

Please select the one below that is the best fit for your research. If you are not sure, read the appropriate sections before making your selection.

☒ Life sciences ☐ Behavioural & social sciences ☐ Ecological, evolutionary & environmental sciences

For a reference copy of the document with all sections, see nature.com/documents/nr-reporting-summary-flat.pdf

Life sciences study design

All studies must disclose on these points even when the disclosure is negative.

Sample size	Representative data and replicates as indicated are shown. Experiment sample size was n=2 or higher. All replicates show same results. No statistical method was used to predetermine sample size.
Data exclusions	Cells that either contained a nucleus with an unusual shape, that did not contain a compact nucleolus, or that contained gigantic vesicles were excluded from the analyses. We considered these cells as unhealthy/apoptotic/dead.
Replication	Data shown are reproducible. Two or more independent replicates are shown and indicated in the figure legends.
Randomization	The experiments were not randomized. Cells that were considered as unhealthy/apoptotic/dead were not imaged.
Blinding	The investigators were not blinded to allocation during experiments and outcome assessment. Person that did the transfection experiment also did the imaging, and therefore was not blinded.

Reporting for specific materials, systems and methods

We require information from authors about some types of materials, experimental systems and methods used in many studies. Here, indicate whether each material, system or method listed is relevant to your study. If you are not sure if a list item applies to your research, read the appropriate section before selecting a response.

Materials & experimental systems

n/a	Involved in the study	
☐	☒	Antibodies
☐	☒	Eukaryotic cell lines
☒	☐	Palaeontology and archaeology
☒	☐	Animals and other organisms
☒	☐	Clinical data
☒	☐	Dual use research of concern
☒	☐	Plants

Methods

n/a	Involved in the study	
☒	☐	ChIP-seq
☒	☐	Flow cytometry
☒	☐	MRI-based neuroimaging

Antibodies

Antibodies used	rabbit anti-NOT2 and anti-NOT3 antibodies (raised in the lab of Elmar Wahle, Halle University, Germany); peroxidase-conjugated donkey anti-rabbit secondary antibody was purchased from Cytiva, catalog number: NA934, lot: 17824033
Validation	antibodies were described previously in Jeske et al., 2006, JBC and used in Temme et al, 2010, RNA, too (e.g.); secondary AB was purchased from Cytiva.

Eukaryotic cell lines

Policy information about cell lines and Sex and Gender in Research

Cell line source(s)	Drosophila Schneider 2-R+ lines was obtained from the lab of Aurelio Teleman, German Cancer Research Center.
Authentication	The cells were not authenticated. They look like typical S2R+ cells.
Mycoplasma contamination	cells were not tested for contamination
Commonly misidentified lines (See ICLAC register)	We are not aware that S2R+ cells are commonly misidentified cells.

Plants

Seed stocks	N/A
Novel plant genotypes	N/A
Authentication	N/A
